# CGALS-YOLO: Vision-Based Sensing for Protective Equipment Wearing Compliance Detection in Underground Environments

**DOI:** 10.3390/s26051646

**Published:** 2026-03-05

**Authors:** Chao Huang, Hongkang Huang

**Affiliations:** School of Computer Science and Technology, Zhejiang University of Science and Technology, 318 Liuhe Road, Liuxia Subdistrict, Xihu District, Hangzhou 310023, China; huangchao422@163.com

**Keywords:** underground, wearing compliance detection, CGAFusion, LSCD

## Abstract

Reliable vision-based sensing of protective equipment wearing compliance is essential for safety monitoring in underground mining environments, where complex lighting conditions, similar background textures, and large variations in the scale of wearable items significantly degrade detection performance. To address these challenges, this study proposes a vision-based protective equipment wearing compliance detection method for underground personnel based on CGALS-YOLO. Traditional object detection models often introduce substantial redundant background information during multi-scale feature fusion, which weakens the perception of key wearing regions, particularly for small-scale targets. To alleviate this issue, a content-guided feature fusion (CGAFusion) module is incorporated into the neck of the YOLOv8 network, enabling adaptive fusion of same-scale multi-path features through the collaborative effects of channel, spatial, and pixel attention mechanisms. This design enhances target-related feature representation while suppressing background interference in complex underground scenes. Furthermore, to reduce parameter redundancy and improve cross-scale discrimination consistency in the detection head, a lightweight shared convolution detection (LSCD) structure is introduced. By employing cross-scale shared convolution parameters, group normalization, and scale-adaptive regression, the proposed model achieves a parameter reduction of approximately 23.9% while lowering computational complexity and maintaining stable multi-scale detection performance. Experimental results on an underground protective equipment wearing compliance dataset demonstrate that CGALS-YOLO improves detection accuracy by approximately 4.6% and recall by 3.1% compared with the baseline YOLOv8n, achieving an mAP@0.5 of 89.4%. These results validate the effectiveness and practical applicability of the proposed method for real-time vision-based safety monitoring in underground environments.

## 1. Introduction

As the coal mining industry progresses from mechanization and automation towards intelligence and unmanned operation, the technical scope of the mine safety guarantee system is continuously expanding. Enhancing the safety of underground operations is one of the important goals of intelligent coal mining [1]. Relevant scholars have further proposed an unmanned intelligent mining system architecture centered on the Internet of Things, advanced sensing, and intelligent control. By establishing an integrated control system of perception, decision making, and execution, they aim to achieve comprehensive monitoring and intelligent regulation of the underground working environment and equipment operation status [2].

However, in the highly intelligent production mode, personnel still need to participate in tasks such as inspection and maintenance. The safety of their actions directly affects the safety of the mine operation. In recent years, to address the problem of supervision of underground personnel, researchers have introduced AI video intelligent recognition technology to automatically analyze the operation videos, enabling intelligent supervision of violations in wearing equipment, and have achieved good application results in actual mines [3], and have shown strong advantages in terms of real-time performance and accuracy [4]. Xu Kai et al. [5] improved the research on the safety helmet wearing recognition task based on the YOLOv3 algorithm by adding feature maps to enhance the ability to detect small targets and combining K-means clustering to optimize the prior anchor box settings, while introducing GIoU loss and focal loss to alleviate the problem of unbalanced positive and negative samples. Wang Yuanbin et al. [6] addressed the issues of insufficient lighting in underground environments, dust interference, and small target sizes of safety helmets by introducing the CBAM attention mechanism to enhance the feature expression of the target area and adding a P2 small target detection layer to improve the multi-scale feature perception ability while adopting the EIoU loss function and ShuffleNetV2 lightweight convolution structure, ensuring detection accuracy while effectively reducing model complexity. He Xueming et al. [7] used the YOLOv8 algorithm with a more lightweight structure and better performance to build a coal mine safety helmet wearing detection model, trained and verified it on the actual underground operation scenario dataset, and the model detection accuracy reached 94.4%, well meeting the application requirements of the automatic detection of safety helmets in underground coal mines. Bai Peirui et al. [8] proposed the DS-YOLOv5 model, combining the improved deep SORT multi-target tracking with the transformer structure and BiFPN feature fusion, effectively improving the robustness and accuracy of safety helmet detection in video scenes under conditions of occlusion and scale changes. Chen Wei et al. [9] addressed the problem of abnormal actions of personnel in underground coal mines under low illumination and strong interference environments and, based on YOLOv8l, introduced the receptive field attention convolution and efficient multi-scale attention mechanism, taking into account detection accuracy and real-time performance, significantly improving the feature focusing ability of the model in complex conditions. Luo Jinjin et al. [10] proposed the YOLOv8-ECW model based on YOLOv8n through the collaborative optimization of multi-scale convolution modules and improved loss functions and achieved high-precision and real-time detection of underground personnel behaviors, further verifying the application potential of the YOLOv8 series models in mine scenarios. Regarding the problem of large-scale and morphological changes of safety helmet targets and easily missed detection, Feng Peiyun et al. [11] proposed an improved Cascade R-CNN safety helmet detection algorithm by introducing deformable convolution and a cascaded detection structure to enhance feature expression and sample discrimination ability, and combining an improved feature pyramid and Soft-NMS post-processing strategy to improve multi-scale detection performance; however, the overall structure of this method is relatively complex and the computational cost is large, and there are still certain limitations in applications with high requirements for speed and lightweight in real-time monitoring and other scenarios.

Although the existing research has made certain progress in the accuracy and robustness of safety helmet detection, there are still deficiencies in the stable identification of protective equipment wearing compliance of personnel in complex mine environments. Affected by factors such as insufficient underground lighting, dust interference, and equipment obstruction, the targets of safety protection equipment in video images have a small scale and unclear detailed features, which are prone to confusion with the background. Especially in the identification of the wearing status of key protective equipment such as safety helmets and self-rescue devices, the problems of missed detection and false detection are particularly prominent. To address these issues, this paper focuses on the identification of protective equipment wearing compliance of underground personnel, starting from the requirements of small target detail enhancement and lightweight real-time application. A lightweight and efficient CGALS-YOLO model is proposed to achieve precise detection and recognition of safety helmet and self-rescue device wearing conditions in order to improve the detection performance of protective equipment wearing compliance of personnel in complex mine scenarios.

## 2. Materials and Methods

### 2.1. The Principle of Yolov8 Algorithm

**Figure 1:**
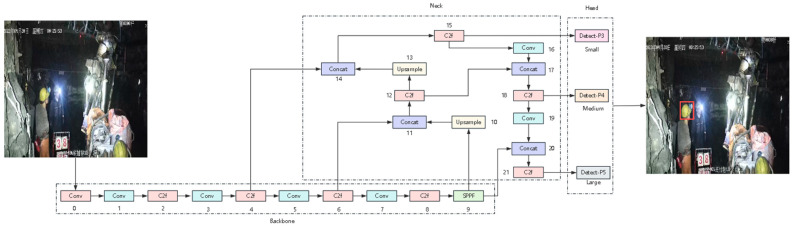
The overall architecture of YOLOv8 and an example underground scene used as the input for detecting protective equipment wearing states.

The YOLO series algorithms belong to the representatives of single-stage object detection methods. Since their proposal, they have been continuously improved, with continuous optimizations in detection accuracy, real-time performance, and model structure design. From the early version of YOLOv1 [12] to YOLOv3 [13], which introduced multi-scale prediction and anchor box mechanism, YOLOv5 [14], which continuously improved in network structure and training strategies, and YOLOv7 [15], the YOLO series has been widely verified in engineering applications and academic research. YOLOv8 [16] is a new generation of object detection model launched by Ultralytics. It has further optimized the network structure, feature extraction method, and training strategy, using a more flexible and efficient module design. It ensures detection accuracy while also taking into account the lightweighting and inference speed of the model, making it suitable for real-time object detection tasks in complex scenarios. The network structure of YOLOv8 mainly consists of backbone, neck, and detect head. Using it as the baseline detection model for subsequent research, its overall network structure is shown in Figure 1.

Among them, the backbone part of Yolov8 is based on the CSP [17] concept and is composed of 5 convolutional modules, 4 C2f modules, and 1 SPPF module. The shallow convolutional modules are mainly responsible for extracting low-level semantic information such as the edges and textures of the target. The intermediate layers are enhanced by stacking multiple C2f modules, gradually improving the analysis ability for the target structure and semantic features. The high-level features are rich in semantic information while maintaining the necessary spatial resolution. The SPPF module introduced at the end uses spatial pyramid pooling operations to expand the receptive field of the network. The Yolov8 neck part, inspired by PANet [18], uses the PAN-FPN structure to achieve the self-top-down and self-bottom-up fusion of the multi-scale p3, p4, and p5 feature outputs by the backbone part. This fusion provides effective feature support for the subsequent detection heads to detect targets at different scales. Compared with the previous version with significant changes, the detection head adopts a decoupled head structure to handle the target classification and bounding box regression separately. In the loss function design, binary cross-entropy loss is used for target class prediction, and CIoU [19] and distribution focus loss DFL [20] are introduced for the bounding box regression task. This approach can effectively reduce the parameter quantity and computational complexity compared with the single detection head structure of previous versions (v3–v5). It no longer relies on the anchor-based prediction method but switches to the anchor-free prediction method, avoiding the dependence on the prior anchor box size, reducing the cost of hyperparameter design and optimization, and enabling the model to have better generalization ability for targets of different scales.

### 2.2. CGAFusion-Based Feature Fusion Module

The working environment in underground operations is characterized by complex light conditions, similar background textures, and significant differences in target sizes. On one hand, due to the small space of the tunnels and poor lighting conditions, there are problems such as uneven brightness, low contrast, and noise interference in the images. On the other hand, the colors of underground equipment, pipelines, and personnel clothing are similar, which can easily cause confusion between the background and the target. Because the target of miners’ protective equipment wearing state has a large scale change in the image, and the detailed information of small targets is easily weakened during the deep feature extraction and multi-scale fusion process, etc., if the model only relies on the traditional feature stitching method in the feature fusion stage, it is prone to introduce a large amount of redundant background information and weaken the ability to focus on the key target areas.

To further enhance the analysis ability of key information in the multi-scale feature fusion stage of YOLOv8, this chapter adopts the idea of CGAFusion (content-guided attention fusion) in the neck part of the YOLOv8 network for the adaptive fusion of features from different paths but with the same spatial scale. CGAFusion is based on the CGAFusion proposed in DEA-Net [21], the content-guided attention fusion idea, which can effectively utilize the effective semantic information in multi-scale features, solve the problem of a large amount of redundant background information caused by traditional feature concatenation methods, and improve the discriminative power of feature representation.

**Figure 2:**
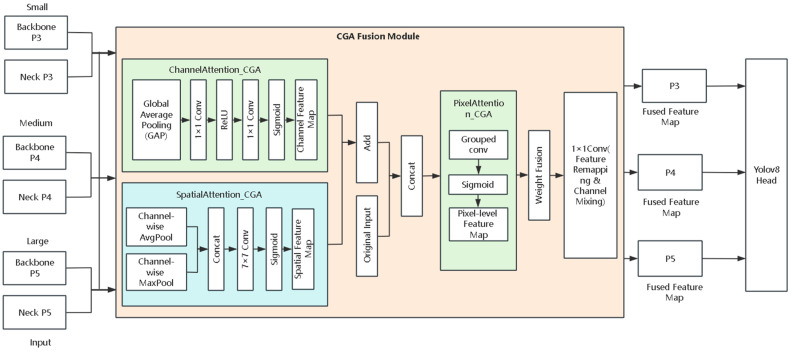
Architecture of the CGAFusion module illustrating channel, spatial, and pixel attention mechanisms.

The specific process is as follows: Firstly, the shallow features Fx output by the backbone network of YOLOv8 and the deep semantic features Fy obtained by the self-top-down path of the neck are aligned on the same scale. Then, these two features are input together into the CGAFusion module for fusion. For the two sets of features of each scale in the feature pyramid, content-guided fusion is performed on the corresponding two sets of features at the P3, P4, and P5 layers. P3 focuses on small target detection, P4 focuses on medium targets, and P5 focuses on large targets. The pixel-level attention weights obtained by CGA adaptively highlight the key regions related to the target, effectively suppressing background interference, thereby enhancing the collaborative expression ability between the shallow detail features and the deep semantic features. This mechanism can improve the model’s robustness in complex environments and also enhance the model’s perception ability for small targets. It is not a single attention mechanism but a fusion strategy framework. Channel attention [22], spatial attention [23], and pixel attention [24] are the three attention sub-modules used to guide content within CGAFusion, which guide and weight the features from the channel, spatial, and pixel levels respectively. The overall process of CGAFusion is shown in Figure 2.

The first attention module is the channel attention module (ChannelAttention_CGA). It first performs global average pooling on the input (aligned) feature maps to obtain the global semantic representation of each channel. Then, it compresses and reconstructs the channel features through a nonlinear mapping structure composed of two 1 × 1 convolutions. The intermediate channel dimensions are compressed according to the ratio reduction = 8, thereby generating attention responses of channel dimensions while reducing computational complexity. These attention responses are used to depict the importance of different channels in the current content, providing semantic guidance information for subsequent pixel-level fusion.

The second attention module is the spatial attention module (SpatialAttention_CGA). This module performs average pooling and max pooling on the input features along the channel dimension, obtaining two complementary feature maps with spatial information. Then, the two feature maps are concatenated and passed through a 7 × 7 convolution to obtain a spatial response map, which represents the correlation of different spatial positions with the target detection task. Different from the traditional spatial attention that directly weights the features, the spatial response map generated by SpatialAttention_CGA does not directly operate on the feature mapping. Instead, it serves as a prior guidance for the spatial dimension and, together with the channel attention, constrains the generation process of pixel-level attention, thereby achieving more detailed feature fusion.

The third attention module is pixel attention (PixelAttention_CGA). This module uses the initial fused features as the content carrier, and combines the results of channel attention and spatial attention to form the guiding information (content guidance). After alignment along the channel dimension, the guiding information and the content features are concatenated, and lightweight grouped convolution is used to generate pixel-level fusion weights. This achieves the purpose of adaptively adjusting the contribution of multi-scale features at different pixel positions. The weights will change with the variations in spatial and channel dimensions, enabling the model to dynamically select the role of the input features at each pixel, retaining and preserving the relevant information related to the target at a fine-grained level, and suppressing redundant background.

After obtaining the fusion weights at the pixel level, the CGA module further performs weighted fusion of the input features from different scales based on these weights. Specifically, at each pixel position, the pixel attention weights are used to dynamically balance the contributions of the shallow detail features and the deep semantic features, and it is combined with the initial fusion features to form the final output features, thereby achieving adaptive fusion of multi-scale features. The formula is expressed as follows.

(1) $$F_{out}=F_{initial}+P⊙F_{x}+(1-P)⊙F_{y}$$

where *F_initial_* = *F_x_* + *F_y_*, and *P* represents the pixel-level attention weight. The symbol ⊙ denotes element-wise multiplication.

Finally, the features after pixel-level weighted fusion processing will be sent to a 1 × 1-sized convolution layer for feature remapping and channel mixing to obtain the final fusion result. This content-guided fusion strategy, on the basis of ensuring that the features on each path are at the same scale and aligned, adaptively selects the key information in each path, largely suppressing the interference caused by redundant backgrounds and also improving the ability to collaboratively analyze shallow detail features and deep semantic features. In the detection head of YOLOv8, the three sets of multi-scale features P3, P4, and P5 will first undergo fusion processing through the CGAFusion module, and then be input into the detection head, thereby achieving the accurate prediction of various targets of different scales.

### 2.3. LSCD-Based Detection Head Optimization

In the task of detecting the protective equipment wearing states of underground miners, there are subtle differences in appearance among the target categories (such as wearing/not wearing safety helmets, self-rescue devices, etc.), and the scale range of the targets is also large. This places higher demands on the discriminative ability and scale adaptability of the detection head. However, the original detection head of YOLOv8 predicts in the multi-scale branches using an independent convolution structure. Although it has good detection performance, it has relatively high parameter scale and computational cost, and the consistency of feature modeling among different scale branches is insufficient, which is not conducive to efficient deployment in resource-constrained scenarios.

**Figure 3:**
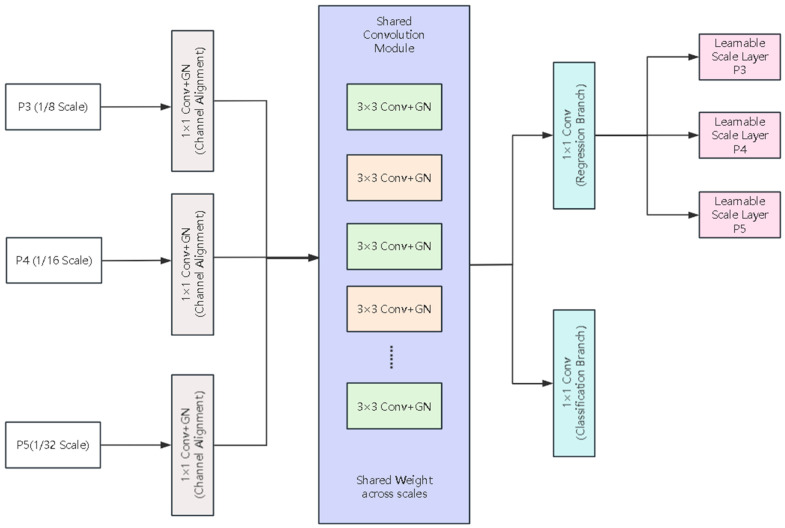
LSCD detection head architecture with lightweight shared convolution for multi-scale prediction.

In response to the aforementioned shortcomings, this chapter, based on the original multi-scale prediction structure of YOLOv8, introduces the LSCD (lightweight shared convolutional detection) structure with lightweight shared convolutional heads. It improves the original detection head structure in terms of lightweighting and enhanced discriminative ability. This enables the model to maintain detection accuracy while effectively reducing the number of parameters and computational complexity. The structure is shown in Figure 3.

Inside the detection head, first, three layers of features, namely P3 (1/8), P4 (1/16), and P5 (1/32), are aligned through lightweight 1 × 1 convolutions to provide consistent feature dimensions for subsequent unified analysis. Then, features of various scales are input into the shared convolution module for feature transformation. This shared convolution module consists of multiple layers of 3 × 3 convolutions and can reuse the discriminative feature extraction ability on different scale branches, avoiding each detection head to repeatedly learn similar semantic patterns, thereby reducing the redundancy of model parameters.

Due to the small sample size and small batch size in the underground environment, the LSCD detection head uses group normalization [25] instead of the traditional batch normalization [26] in the convolutional structure. This normalization method does not require statistical characteristics in the batch dimension and can maintain a stable feature distribution even during small batch training, which is beneficial for improving the convergence stability and generalization performance of the model.

To adapt to the scale differences of different scale targets in the bounding box regression space (i.e., the variation in object sizes in images, such as small distant targets versus large nearby targets), LSCD introduces independent learnable scale layers in each scale detection branch to adaptively scale the output of the regression branch. This design maintains the sharing of convolution parameters while enabling different scale features to dynamically adjust the regression response according to the size differences of the targets, effectively alleviating the optimization difficulties caused by the inconsistent distribution of multi-scale targets in the regression space, and thereby improving the training stability and overall robustness of the model in tasks of detecting small and large targets.

### 2.4. CGAFusion–LSCD Joint Optimization Module

In order to fully utilize the respective advantages of the two modules, this chapter introduces the CGAFusion module at the neck and detection head of the YOLOv8 network structure, and the LSCD detection head structure at the detection head end, and integrates the two to construct an improved network structure for the task of detecting wearing compliance in underground environments, which is named CGALS-YOLO.

**Figure 4:**
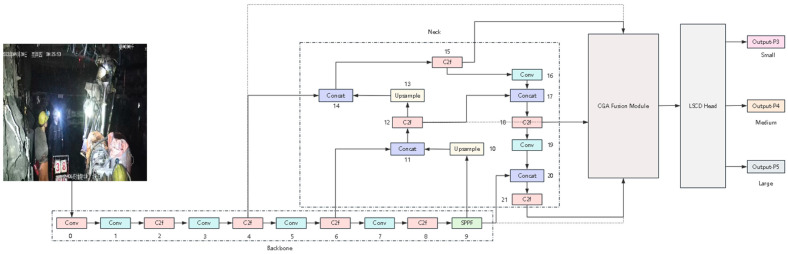
Overall architecture of CGALS-YOLO integrating CGAFusion in the neck and LSCD in the detect head.

At the neck end, the quality of feature fusion is strengthened, and at the detection head end, the discrimination efficiency and scale adaptability are improved. CGALS-YOLO simultaneously enhances the feature expression ability and detection performance without significantly increasing the model complexity. Figure 4 shows the entire network structure diagram.

## 3. Experimental Results and Analysis

To verify the effectiveness of the proposed underground personnel protective equipment wearing compliance recognition method based on CGALS-YOLO, this paper conducts a series of comparative experiments and performance evaluations on the constructed dataset. The experimental results are analyzed from aspects such as overall recognition performance, recognition effects of different wearing categories, and model real-time performance.

### 3.1. Dataset Construction and Evaluation Metrics

High-quality datasets are the foundation for the development of a personnel compliant wearing recognition system in underground coal mines. Due to the closed environment and complex working conditions in underground coal mines, the existing public datasets still have certain deficiencies in terms of scene coverage and environmental diversity, making it difficult to fully reflect the actual working conditions in the real mining area.

**Figure 5:**
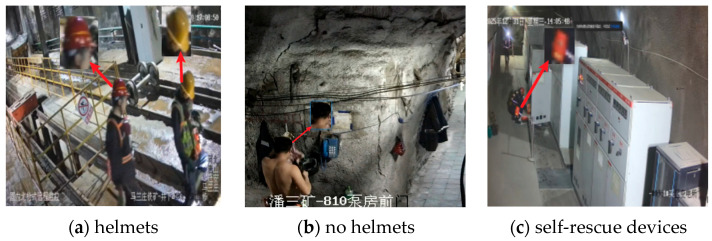
Representative samples of the constructed underground safety wear dataset. (**a**) Personnel wearing safety helmets; (**b**) personnel without safety helmets in real working or monitoring environments; (**c**) personnel equipped with self-rescue devices. These examples highlight the visual differences and environmental variations among categories.

To enhance the diversity and applicability of the dataset, this paper comprehensively selected publicly available underground datasets such as DsDPM 66 [27], CUMT-HelmeT [28], and DsLMF+ [29], and combined them with the sampled data collected from on-site monitoring cameras in the mining area. Through the method of multi-source data fusion, a comprehensive dataset for identifying protective equipment wearing compliance in underground coal mines was constructed to achieve better generalization performance of underground data. The target categories covered in the dataset are shown in Figure 5, including typical underground personnel wearing states such as Figure 5a wearing safety helmets, Figure 5b not wearing safety helmets, and Figure 5c wearing self-rescue devices, which are used to support the training and evaluation of subsequent protective equipment wearing detection models. For clearer visualization, each subfigure additionally provides a zoomed-in view of the key regions of interest, indicated by red arrows, to highlight the discriminative features used by the model.

All human subjects’ faces in the collected images were anonymized by blurring or masking to protect privacy. The data collection process was conducted in accordance with institutional guidelines, and informed consent was obtained from personnel where required.

This paper collected 4832 images of underground mining operation scenes, including 1450 images without safety helmets, 2362 images with safety helmets, and 1020 images with self-rescue devices. The dataset was divided into training, validation, and test sets at a ratio of 7:2:1, ensuring that each split preserved scene diversity and minimized overlap between similar scenes. For model training and evaluation, all input images were resized to 640 × 640 pixels. Various data augmentation techniques provided by the YOLOv8 framework were applied, including random horizontal flipping, scaling, rotation, brightness and contrast adjustments, and noise addition, which improve model robustness under diverse underground scenarios. To mitigate potential biases arising from the multi-source dataset, we ensured a balanced representation of each target category across different sources. Specifically, we carefully sampled images from each dataset to avoid overrepresentation of any particular environment or lighting condition. These measures helped improve the generalization ability of the model to unseen underground environments and reduced the risk of overfitting to specific sources.

To comprehensively evaluate the detection performance of the underground miner safety wearing status recognition model that was constructed, this chapter selects six indicators as the evaluation criteria for the model’s performance, namely accuracy (precision, P), recall (recall, R), average precision (AP), mean average precision (mAP), model parameter quantity (Params), computational quantity (FLOPs), and detection speed (frames per second, FPS).

In the task of detecting the compliance of miners’ safety equipment usage, the model mainly identifies three targets: wearing a safety helmet, not wearing a safety helmet, and wearing a self-rescue device. The core of the evaluation is the four types of prediction results, namely TP (correctly predicted as the target), TN (correctly predicted as non-target), FP (incorrectly predicted as the target), and FN (incorrectly predicted as non-target).

Among them, accuracy is used to measure the reliability of the model’s prediction for positive samples, while recall is used to measure the model’s ability to identify positive samples; AP is quantified by the area under the precision–recall curve to measure the detection accuracy of a single class target; mAP is the average of the AP values for all classes, and can be further divided into mAP50 and mAP50-95. mAP50 represents the mAP when the IoU threshold is 0.5, and mAP50:95 represents the average mAP within the range of IoU thresholds from 0.5 to 0.95. These two are used to measure the detection performance at different IoU thresholds. Params and FLOPs represent the complexity and computational burden of the model; FPS represents the number of frames detected per second and is used to measure the detection speed of the model.

The formula is expressed as follows:

(2) $$P=\frac{TP}{TP+FP}$$

(3) $$R=\frac{TP}{FP+FN}$$

(4) $$AP=\int_{0}^{1}P(R)dR$$

(5) $$mAP=\frac{1}{C}\sum_{c=1}^{C}AP_{c}$$

where, in this article, C = 3, corresponding to three types of targets (wearing safety helmets, not wearing safety helmets, wearing self-rescue devices).

### 3.2. Experimental Environment

To ensure the reproducibility of the experimental results and the fairness of the comparative analysis, all experiments were conducted under the same software and hardware environment, as shown in Table 1. All results correspond to the average performance over three independent training runs with identical hyperparameters. The experimental platform’s hardware environment uses the Intel Core i5-8300H processor with a frequency capable of meeting the basic requirements for training and inference of deep learning models. The GPU is NVIDIA GeForce GTX 1050 Ti, which has parallel computing capabilities and can support the training and testing of target detection models. The system memory is 16 GB, which can meet the requirements for data loading and memory during model operation. The operating system is Windows 10 (64-bit), the deep learning framework is PyTorch 1.13.1, the programming language is Python 3.8, and the model training and experimental management are carried out in the PyCharm 2024.3.5 development environment. The related deep learning dependent libraries and running environment are kept consistent to prevent differences in experimental results caused by different environment configurations.

### 3.3. Experimental Algorithm Results

**Figure 6:**
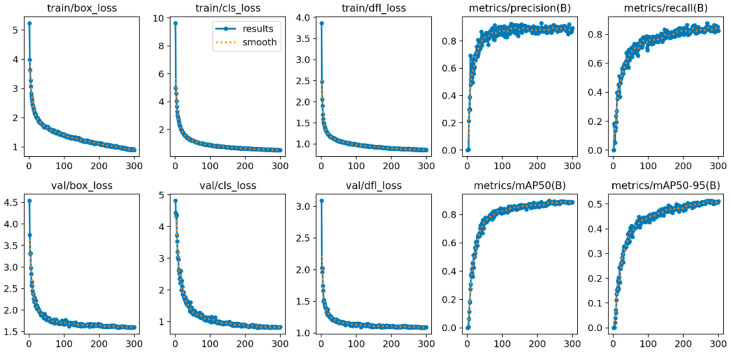
Convergence curves of various indicators of the improved model.

The improved CGALS-YOLO algorithm was used to train and test on the self-built underground compliance wearing dataset, and YOLOv8n was used as the baseline model for comparative experiments. Figure 6 shows the convergence of various indicators of the model during the training process, including the confidence loss, bounding box regression loss, and classification loss curves of the training set and validation set, as well as the trends of performance evaluation indicators such as precision, recall, mAP@0.5, and mAP@0.5:0.95 with the number of training epochs.

From the loss function curve, it can be observed that CGALS-YOLO is stable and convergent during the training process, and all the loss values are at a relatively low level. Among them, the bounding box regression loss fluctuates within a small range overall, the confidence loss shows a downward trend, and the classification loss gradually approaches 0 after approximately 200 epochs, indicating that the model has successfully learned the discriminative features of the target category.

**Figure 7:**
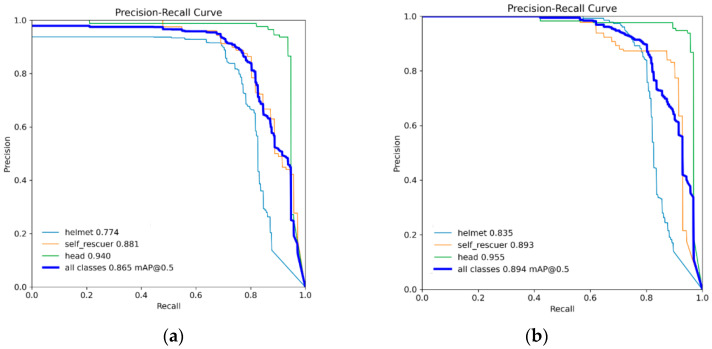
Precision–recall curves of the baseline model YOLOv8n (**a**) and the proposed CGALS-YOLO model (**b**).

**Figure 8:**
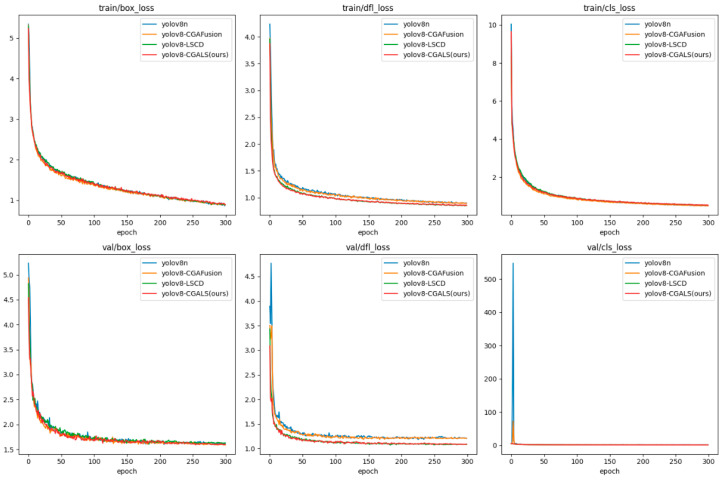
Comparison chart of training loss curves.

**Figure 9:**
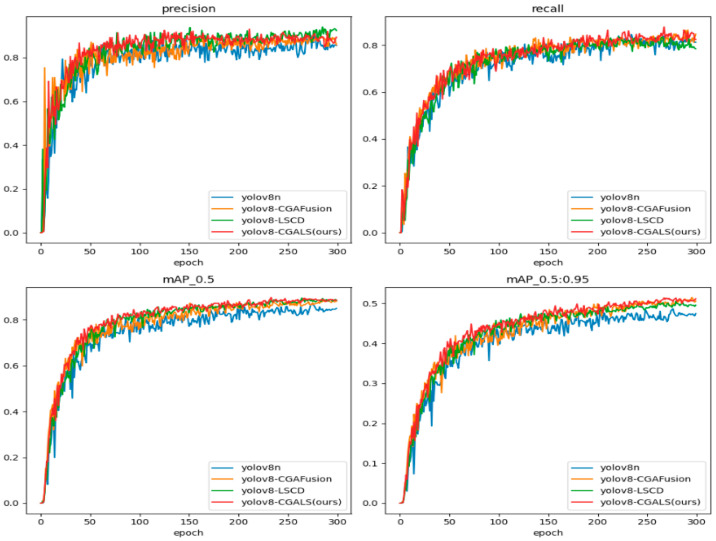
Comparison chart of various indicator curves.

**Figure 10:**
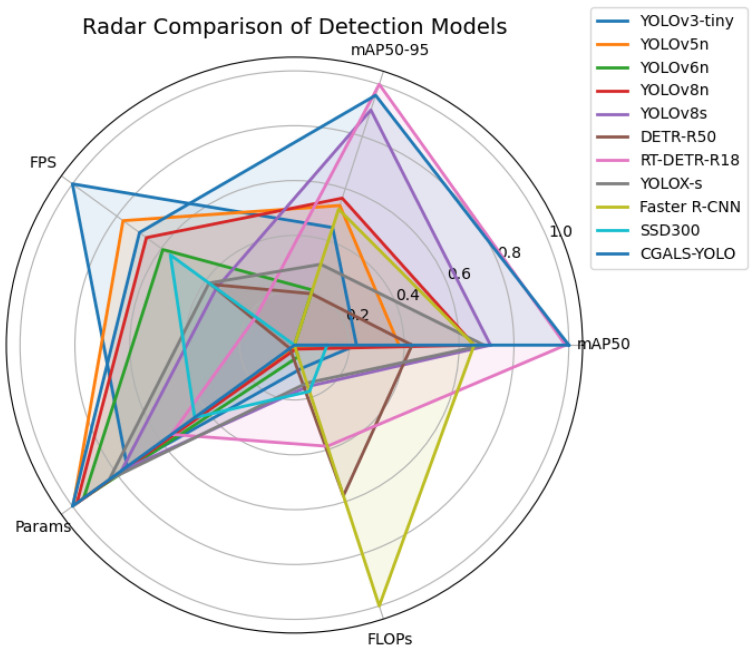
Radar chart comparison of representative detectors across multiple evaluation dimensions. The chart illustrates that classical and modern models exhibit different trade-offs, supporting the comprehensiveness of the selected baselines.

To further analyze the detection performance of the improved algorithm on different target categories, the P–R curves of the baseline model YOLOv8n and the improved model CGALS-YOLO were compared on the self-built test set. The results are shown in Figure 3, Figure 4, Figure 5, Figure 6, Figure 7, Figure 8, Figure 9 and Figure 10. From the figure, it can be seen that, compared with YOLOv8n, CGALS-YOLO has a larger envelope area of the overall precision–recall curve in the three target categories of helmet, self_rescuer, and head. It still maintains high accuracy in the medium and high recall rate range, indicating that the proposed method has better stability and robustness for detecting in complex underground environments.

Table 2 shows that the improved CGALS-YOLO outperforms YOLOv8n by 93.1% in helmet-type object detection. For complex maintenance environments requiring manual intervention, the false detection rate has decreased. For head-type objects, the model has improved detection stability while maintaining a high recall rate. For self_rescuer targets with large scale variations and prone to occlusion, CGALS-YOLO has significantly improved in mAP@0.5 and mAP@0.5:0.95, indicating that the improved algorithm has better adaptability to small targets and complex backgrounds. Overall, CGALS-YOLO has improved in all four indicators: precision, recall, mAP@0.5, and mAP@0.5:0.95, indicating that the proposed method can enhance detection accuracy and the robustness of the model.

### 3.4. Ablation Experiment and Result Analysis

To verify the effectiveness of adding the CGAFusion and LSCD modules, this paper conducted multiple ablation comparison experiments on a self-built security wear dataset. The results are shown in Table 3. The symbol “+” indicates that the corresponding improved module was added to the model. All experiments were carried out using the same training strategy, with the training period uniformly set to 300 epochs, and YOLOv8n was used as the benchmark model.

From the results of the ablation experiments, it can be seen that when only the CGAFusion module is added to the baseline model YOLOv8n, mAP@0.5 and mAP@0.5:0.95 increase by 1.8% and 3.1% respectively. From these results, it can be concluded that using a content-guided attention mechanism can enhance multi-scale feature fusion, enabling the model to pay more attention to and focus on the areas related to safe wearing, thereby improving the detection performance.

To further understand how CGAFusion enhances performance, we analyzed its behavior from a problem-oriented perspective. The characteristics of the underground environment are complex lighting and chaotic backgrounds, which often reduce the representation effect of small protective equipment targets. Channel attention emphasizes informative semantic channels and enhances sensitivity to subtle appearance cues under low-contrast conditions. Spatial attention introduces spatial priors, which helps to suppress interference from similar textures and background structures. However, when each attention component is applied alone, the improvements they provide are complementary and limited. The main performance improvement comes from their collaborative interaction within the CGAFusion framework. Specifically, the pixel attention module performs fine adaptive fusion under the guidance of spatial and channel cues, and can more effectively retain the details of small targets during multi-scale feature interactions. Through this collaborative mechanism, CGAFusion improves feature discrimination ability and brings continuous improvements in accuracy and mean average precision (mAP).

Only by incorporating the LSCD detection head into YOLOv8n, the experimental results show that the model’s parameter quantity has decreased by 24.8%, FLOPs have reduced by 24.1%, but mAP@0.5 has increased by 1.7%. This indicates that the lightweight shared convolutional detection head not only ensures the detection accuracy but also significantly reduces the model complexity. However, it has an impact on the recall metric, suggesting that lightweighting the detection head alone may slightly reduce the target recall capability in complex scenarios.

When the CGAFusion and LSCD modules are fused together to form the CGALS-YOLO model, the experimental results show that the model outperforms the baseline model in terms of precision, recall and mAP metrics. Recall has increased by 4.6%, mAP@0.5 has increased by 2.9%, and mAP@0.5:0.95 has increased by 2.8%. At the same time, the model’s parameter quantity and computational cost have decreased by approximately 20.0% and 20.7% respectively. Thus, it can be seen that CGAFusion enhances discriminative ability during the feature fusion stage, while effectively compensating for the decrease in recall caused by the lightweighting of LSCD. The two have formed a good complementary relationship in terms of performance and complexity.

In order to better analyze the impact of each improvement module on the model training process and performance changes, a comparative analysis was conducted on the training loss curves and detection performance indicators of the four models: YOLOv8n, YOLOv8n + CGAFusion, YOLOv8n + LSCD, and CGALS-YOLO. The results are shown in Figure 8 and Figure 9 respectively.

Figure 8 shows the changes in loss for the four models during the training and validation phases, namely train/box_loss, train/dfl_loss, train/cls_loss, and their corresponding val/box_loss, val/dfl_loss, val/cls_loss. From the overall trend, it can be observed that the loss functions of the four models gradually decrease and converge as the number of training rounds increases, indicating that the training strategy used is relatively stable. CGALS-YOLO converges faster in most loss items and has a smaller final loss value.

Figure 9 shows the changes in precision, recall, mAP@0.5, and mAP@0.5:0.95 during the training process of the four models. From the figure, it can be seen that, as the number of training rounds increases, the detection performance of each model gradually improves and stabilizes. CGALS-YOLO significantly improves in indicators such as recall and mAP, maintaining a leading position or approaching the optimal level in most training stages, and ultimately achieving the best comprehensive performance in mAP@0.5 and mAP@0.5:0.95.

The comprehensive analysis of the ablation experiment results proves that the CGAFusion and LSCD modules are effective and reasonable in the task of safe wear target detection. The proposed CGALS-YOLO model achieves a comprehensive improvement in performance and accuracy while maintaining a low computational cost.

### 3.5. Experimental Comparison and Result Analysis

To further verify the comprehensive performance of the proposed CGALS-YOLO model in terms of detection accuracy, real-time capability, and model complexity, this paper conducted comparative experiments with several mainstream object detection models under the same experimental environment (all comparison models were trained or fine-tuned on the proposed dataset using a unified training strategy to ensure fairness) and dataset conditions. These models include YOLOv3-tiny, YOLOv5n, YOLOv6n [30], YOLOv8n, YOLOv8s, DETR-R50 [31], RT-DETR-R18 [32], YOLOX-s [33], Faster R-CNN (FPN) [34], and SSD300 (VGG16) [35]. The selected methods represent different detection paradigms and development stages: YOLOv3-tiny and SSD300 serve as classical lightweight real-time baselines, YOLOv5/YOLOv6/YOLOv8 and YOLOX represent the evolution of one-stage detectors, DETR-R50 and RT-DETR-R18 correspond to transformer-based detection frameworks, and Faster R-CNN (FPN) represents the classical two-stage detection paradigm. This diverse selection enables a comprehensive evaluation of the proposed method across accuracy, efficiency, and model complexity. The experimental results are shown in Table 4.

To further analyze the characteristics of representative detectors beyond a single accuracy metric, a radar chart comparison (Figure 10) is provided, covering multiple factors such as detection accuracy, model complexity, inference speed, robustness, and generalization ability. The visualization illustrates the complementary strengths of classical CNN-based, lightweight, and transformer-based detectors, indicating that these baselines still provide meaningful and diverse reference points rather than being obsolete.

From the perspective of detection accuracy indicators, CGALS-YOLO achieves the best or nearly best performance in core evaluation indicators such as precision, recall, and mAP. Among them, the model’s precision reaches 91.3%, recall reaches 85.1%, and mAP@0.5 and mAP@0.5:0.95 reach 89.4% and 51.3% respectively. The overall detection performance is outstanding.

Compared with the benchmark model YOLOv8n, CGALS-YOLO improves by 2.9% and 2.8% in the mAP@0.5 and mAP@0.5:0.95 indicators respectively. It has obvious advantages in positioning accuracy, target recognition degree, and stability of multi-scale detection.

The lightweight model YOLOv3-tiny has certain advantages in inference speed, but the recall and mAP indicators are significantly lower. It is prone to missing detections in complex underground scenarios and cannot meet the actual requirements of accuracy and reliability for safety wear detection. Compared with medium-sized and large models, the overall detection performance of CGALS-YOLO is superior to models such as YOLOv8s, YOLOX-s, etc. Although RT-DETR-R18 performs well in the mAP indicator, its parameter size and computational complexity are very large, and it cannot be deployed in real-time in embedded or edge computing environments with limited computing power.

Compared with the classic two-stage detection model Faster R-CNN (FPN) and the single-stage detection model SSD300 (VGG16), Faster R-CNN achieves 86.5% and 48.2%, respectively, in mAP@0.5 and mAP@0.5:0.95. However, its parameter size is as high as 41.7 M, computational complexity reaches 143.8 GFLOPs, and inference speed is only 10.8 FPS, which cannot meet the real-time requirements of underground safety wear detection. SSD300 has good inference speed with an FPS of 55.4, but its detection accuracy is limited, with mAP@0.5 only reaching 82.0%. The detection ability for small-scale and occluded targets in complex underground environments is poor, and it is prone to missing detections.

### 3.6. Visual Comparison and Scale-Specific Analysis

**Figure 11:**
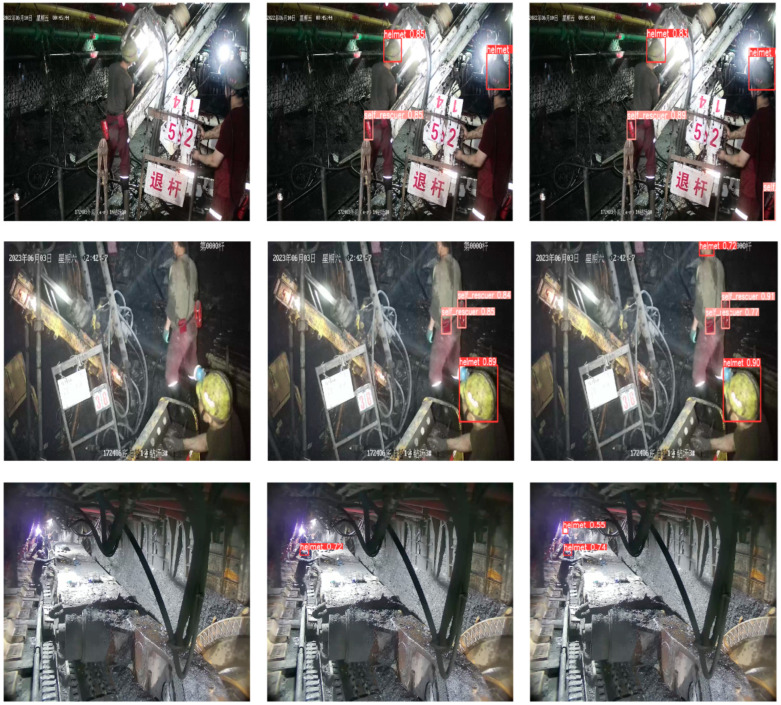

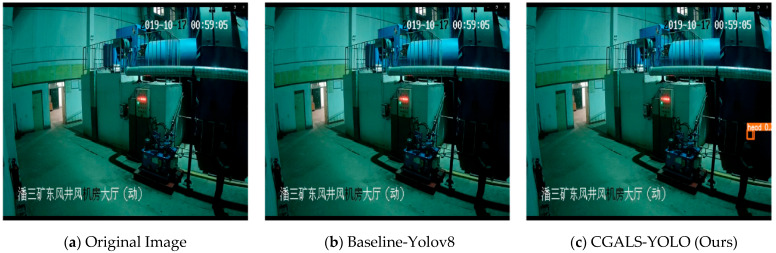
Detection result comparisons on four representative underground monitoring scenes. Each row corresponds to one scene. Columns show (**a**) the original image, (**b**) predictions from the baseline YOLOv8 model, and (**c**) predictions from the proposed CGALS-YOLO model. Zoom-in panels highlight small or occluded targets to illustrate improvements in detection localization and recognition under challenging conditions such as low light, occlusion, and high brightness.

To visually demonstrate the detection differences between the baseline model and the improved model in the task of identifying wearing compliance in underground environments, four representative monitoring scenarios were selected for visual comparison, as shown in Figure 11. In the figure, each row represents a monitoring scenario. From left to right, (a) shows the original image, (b) shows the prediction of the baseline model, and (c) shows the prediction of CGALS-YOLO.

In the first set of scenarios, the self-rescue device target is located at the lower right edge of the image and is of a small size. The baseline model failed to detect this target, while the improved model successfully identified it, indicating that the proposed method has a stronger ability in detecting small targets at image edges.

In the second set of scenarios, the upper right part of the safety helmet of the person is obscured by the image boundary and only a partial area is retained. The baseline model failed to detect the helmet, but CGALS-YOLO was still able to accurately identify it, indicating that the improved model has better robustness under occlusion conditions.

In the third group of scenarios, due to the high brightness of the miner’s lamp causing strong light interference in the head area, the baseline model failed to correctly identify the helmet target, while the improved model successfully detected it. This indicates that the model has a stronger discriminative ability in the presence of strong light and feature degradation.

In the fourth scenario, the overall brightness of the images was extremely low, and the target features were not prominent. Under such poor illumination conditions, the pedestrian’s body contour and head region are difficult to clearly distinguish with the naked eye. The baseline model failed to detect the person’s head, while the improved model successfully identified it. Although the detection confidence was relatively low and a few duplicate detection boxes appeared, the detected region corresponded to the actual head location. The enhanced-illumination comparison further confirmed the correctness of the detection. This example demonstrates the improved model’s stronger robustness and adaptability under extreme low-light conditions.

Based on the above analysis, the proposed CGALS-YOLO model in this paper demonstrates superior detection stability and confidence in complex underground environments, specifically for typical unsafe wearing scenarios such as low light, severe occlusion, small targets, and strong light interference. It can more accurately identify protective equipment wearing compliance of miners, thereby verifying the effectiveness and practical value of this model in underground safety monitoring tasks.

**Figure 12:**
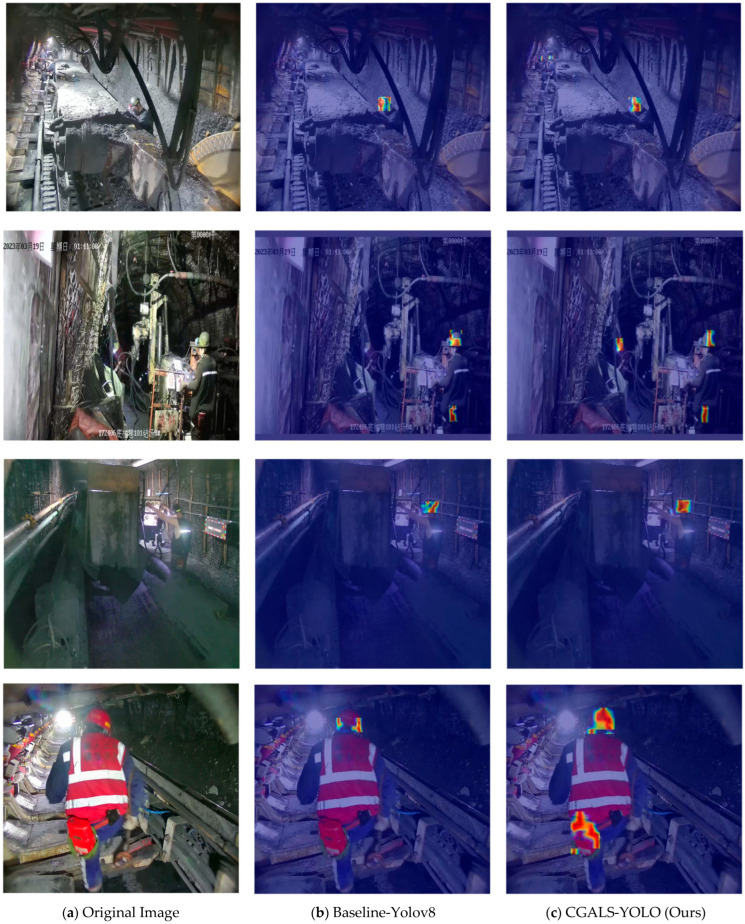
Activation map visualization comparison between the baseline YOLOv8 model and the proposed CGALS-YOLO model. Each row corresponds to one representative underground monitoring scene, and columns show (**a**) the original image, (**b**) activation maps of the baseline model, and (**c**) activation maps of the proposed model. The color bar indicates the normalized activation intensity, where warmer colors (red/yellow) represent higher attention responses and cooler colors (blue) indicate lower responses. The results demonstrate that the proposed model focuses more accurately on protective equipment regions while suppressing background interference, especially under challenging conditions such as low illumination, occlusion, and complex backgrounds.

To further provide visual interpretability and illustrate how the proposed model suppresses background interference while focusing on protective equipment, activation maps were generated using the Grad-CAM method on representative test images. Four typical scenarios corresponding to Figure 12 were selected for comparison between the baseline model and CGALS-YOLO.

In the first scenario, the baseline model mainly activated on the head region of the nearby miner on the right side, while CGALS-YOLO not only focused on this region but also produced clear activation responses on the head areas of two distant miners. This indicates that the proposed model is more capable of capturing small and distant targets.

In the second scenario, the baseline model failed to show activation for the helmet of the left-side miner, whereas the improved CGALS-YOLO successfully detected it. This demonstrates that the improved feature fusion mechanism enhances the model’s sensitivity to protective equipment.

In the third scenario, both models detected the worker, but the activation intensity of CGALS-YOLO was more concentrated and prominent in the head region. This suggests that the proposed model forms more discriminative feature representations under complex illumination conditions.

In the fourth scenario, the baseline model failed to produce meaningful activation on the self-rescuer of a nearby large target, while CGALS-YOLO generated strong and concentrated responses on both the self-rescuer and head regions. This indicates that the model can better localize key semantic regions and reduce background distractions.

Overall, the activation map comparisons demonstrate that CGALS-YOLO consistently produces more focused and semantically meaningful responses on protective equipment regions, while suppressing irrelevant background areas. This provides intuitive evidence that the proposed attention-guided fusion mechanism enhances feature discrimination and contributes to improved detection performance in complex underground environments.

To complement the visual comparison in Figure 11 and Figure 12, we further provide a scale-specific quantitative analysis of detection performance. Table 5 summarizes the average precision (AP) and average recall (AR) of YOLOv8 and CGALS-YOLO for small, medium, and large targets. This table highlights the improvements achieved by CGALS-YOLO, particularly in detecting small targets, which are prone to missing detections in complex underground environments. The results demonstrate that the proposed model not only improves overall accuracy but also enhances fine-grained detection capabilities across different target scales, reinforcing the observations from the visual examples.

### 3.7. Limitations

**Figure 13:**
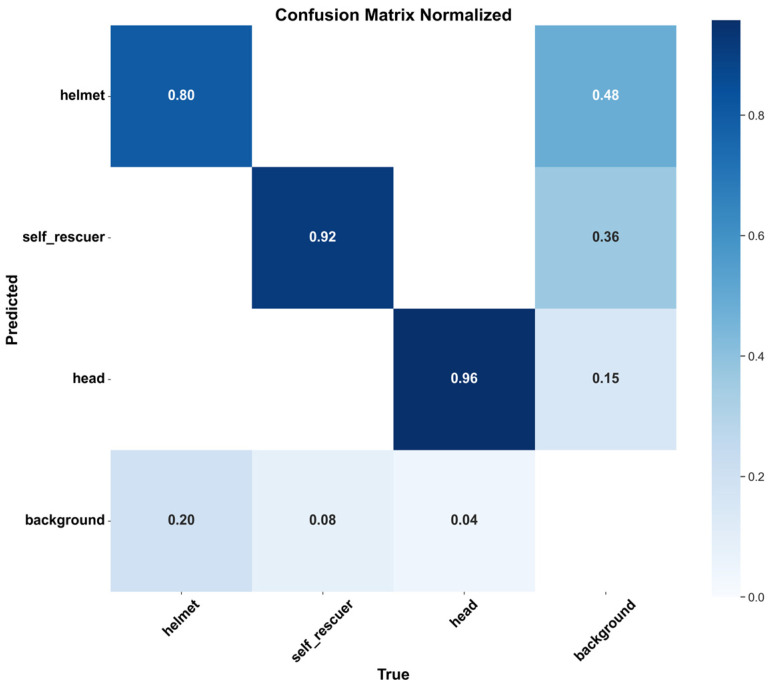
Normalized confusion matrix of CGALS-YOLO on protective equipment detection.

Although the CGALS-YOLO model achieves high detection accuracy for protective equipment, analysis of the normalized confusion matrix (Figure 13) reveals certain limitations.

1. Severe occlusions: Helmet detection shows a notable false negative rate (approximately 48% predicted as background), indicating that occluded personnel or obstructed viewpoints can reduce detection accuracy.
2. Dense dust: Self-rescuer detection also suffers some false negatives (36%), suggesting that poor visibility due to underground dust can affect performance.
3. Camera geometry dependencies: While head detection is generally robust (96% correct), misdetections occur (15% false negatives) in unusual camera angles or perspectives.
4. Hardware and latency considerations: The latency evaluation in this study was conducted on a desktop GPU environment, which may not fully reflect deployment conditions in real industrial systems. In practical underground monitoring scenarios, models are often deployed on embedded or edge devices with limited computational resources. Under such constraints, inference speed may decrease, and additional optimization strategies such as model quantization, pruning, or lightweight deployment frameworks would be required. It is worth noting that CGALS-YOLO itself maintains a relatively lightweight architecture, with only 2.52 M parameters and 6.9 G FLOPs, which is smaller than the YOLOv8 baseline, while achieving improved accuracy. Although we have not yet tested the model on embedded hardware, these characteristics suggest that CGALS-YOLO has strong potential for efficient deployment. Further investigation on embedded platforms will be an important direction for future work to validate its real-time applicability in industrial environments.

These findings highlight that, although the model performs well under typical conditions, its reliability decreases under extreme environmental conditions or unfavorable camera setups.

## 4. Conclusions

This paper addresses the challenges of complex lighting conditions, strong background interference, and large-scale variations of wearable targets in underground coal mines, and proposes a vision-based protective equipment wearing compliance detection method based on CGALS-YOLO. By jointly optimizing feature fusion and detection head design within the YOLOv8 framework, the proposed approach enhances detection robustness and accuracy in complex underground environments, providing effective visual sensing support for underground safety monitoring.

Firstly, in the neck stage, a CGAFusion content-guided feature fusion module is introduced. Through the collaborative modeling of channel attention, spatial attention, and pixel attention, it realizes the adaptive fusion of shallow detail features and deep semantic features, significantly suppressing redundant background interference and enhancing the model’s perception ability for small targets and key wearing areas. Secondly, in the detect head stage, a LSCD lightweight shared convolution detection head is introduced. By parameter sharing, group normalization, and scale adaptive regression mechanism, it reduces the model’s parameter quantity and computational complexity while improving the consistency of multi-scale target discrimination and training stability.

Experimental results demonstrate that CGALS-YOLO achieves superior performance compared with several representative object detection models in underground protective equipment wearing compliance detection, achieving higher detection accuracy, improved real-time efficiency, and a more lightweight model design. These results confirm the effectiveness and practical applicability of the proposed method for vision-based safety monitoring in underground environments. Future work will further integrate temporal information and multimodal sensing data to enhance model generalization and robustness for continuous monitoring under extreme underground conditions.

## Figures and Tables

**Table 1:** Experimental environment and parameter configuration.

Hardware and Software and Training Configuration	Parameter
CPU	Intel Core i5-8300H
GPU	NVIDIA GeForce GTX 1050 Ti
Memory	16 GB
Operating system	Windows10-64 bit
Development tool	PyCharm 2024.3.5
Deep learning framework	Pytorch 1.13.1
Programming language	Python 3.8
Training rounds	300

**Table 2:** Performance comparison between YOLOv8n and experimental algorithms.

Algorithm	Classes	P	R	mAP@0.5	mAP@0.5-0.95
Yolov8n	helmet	0.813	0.752	0.774	0.416
	head	0.943	0.905	0.94	0.512
	self_rescuer	0.844	0.803	0.881	0.528
	all	0.867	0.82	0.865	0.485
Experimental algorithm -CGALS-YOLO	helmet	0.931	0.739	0.835	0.455
	head	0.947	0.942	0.955	0.502
	self_rescuer	0.861	0.873	0.893	0.583
	all	0.913	0.851	0.894	0.513

**Table 3:** Ablation results evaluating the effectiveness of the proposed CGAFusion and LSCD modules on the self-built protective equipment wearing compliance dataset. The baseline model is YOLOv8n, and all experiments were conducted under identical training settings to ensure fairness.

Model	Precision/%	Recall/%	mAP50/%	mAP50:95/%	Params/M	FLOPs/G
Yolov8 (baseline)	86.7	82.0	86.5	48.5	3.15	8.7
Yolov8 + SA_CGA	88.5	81.4	85	49.1	3.09	8.2
Yolov8 + CA_CGA	90.5	77.4	84.1	48.9	3.11	8.2
Yolov8 + SA_CGA + CA_CGA	85.6	77.9	83	48.5	3.12	8.2
Yolov8 + CGAFusion	89.0	82.4	88.3	51.6	3.31	9
Yolov8 + LSCD	92.8	80.1	88.2	50.5	2.37	6.6
CGALS-YOLO (ours)	91.3 (+4.6%)	85.1 (+3.1%)	89.4 (+2.9%)	51.3 (+2.8%)	2.52 (−0.63%)	6.9 (−1.8%)

**Table 4:** Comparative experiment results of various models.

Model	P/%	R/%	mAP50/%	mAP50:95/%	FPS	Params/M	FLOPs/G
Yolov3-tiny	88.2	75.3	82.9	47.7	90.6	12.17	19
Yolov5n	86.3	80.5	84.2	48.3	72.4	2.65	7.7
Yolov6n	85.7	76.4	81.0	46.0	58.1	4.5	13
Yolov8n	86.7	82.0	86.5	48.5	64.0	3.31	9
Yolov8s	87.6	84.2	87.0	50.9	38.9	11.16	28.6
DETR-R50	-	-	84.6	45.9	40.9	40.9	86
RT-DETR-R18	-	-	89.3	51.6	24.7	20	60
Yolox-s	-	-	86.8	46.7	41.5	9.0	26.8
Faster R-CNN (FPN)	-	-	86.5	48.2	10.8	41.7	143.8
SSD300 (VGG16)	-	-	82.0	44.5	55.4	24.1	31.2
CGALS-Yolo (ours)	91.3	85.1	89.4	51.3	66.5	2.52	6.9

**Table 5:** Comparison of AP and AR metrics for different target scales between YOLOv8 and CGALS-YOLO. The table shows average precision (AP) at IoU thresholds 0.50:0.95, 0.50, 0.75 and average recall (AR) for all, small, medium, and large targets. CGALS-YOLO consistently outperforms YOLOv8, especially for small targets, demonstrating improved fine-grained detection performance.

Metric	Area	Baseline-Yolov8	CGALS-YOLO
AP@[0.50:0.95]	All	0.467	0.498
AP@[0.50:0.95]	Small	0.241	0.272
AP@[0.50:0.95]	Medium	0.535	0.552
AP@[0.50:0.95]	Large	0.599	0.613
AP@[0.50]	All	0.851	0.880
AP@[0.75]	All	0.471	0.482
AR@[0.50:0.95]	All (maxDets = 100)	0.556	0.565
AR@[0.50:0.95]	Small	0.349	0.401
AR@[0.50:0.95]	Medium	0.608	0.611
AR@[0.50:0.95]	Large	0.693	0.652

## Data Availability

Publicly available datasets used in this study include DsDPM 66 [27], CUMT-HelmeT [28], and DsLMF+ [29]. The additional data collected from on-site underground monitoring cameras were self-constructed by the authors and are not publicly available due to privacy, security, and ethical restrictions. These data may be made available from the corresponding author upon reasonable request.
